# Antioxidant Potential of *Lycium barbarum* Extracts Is Shaped by Extraction Strategy, Not by Total Phenolic Content Alone

**DOI:** 10.3390/molecules31193441

**Published:** 2026-09-27

**Authors:** Aleksandra Plucińska-Krawczyk, Karolina Miśkiewicz, Justyna Rosicka-Kaczmarek, Alina Kunicka-Styczyńska

**Affiliations:** 1 Department of Sugar Industry and Food Safety Management, Faculty of Biotechnology and Food Sciences, Lodz University of Technology, Wólczańska 171/173, 90-530 Łódź, Poland; alina.kunicka@p.lodz.pl; 2 Institute of Food Technology and Analysis, Faculty of Biotechnology and Food Sciences, Lodz University of Technology, Wólczańska 171/173, 90-530 Łódź, Poland; karolina.miskiewicz@p.lodz.pl (K.M.); justyna.rosicka-kaczmarek@p.lodz.pl (J.R.-K.)

**Keywords:** goji berry, phenolic fingerprint, antioxidant activity, solvent selectivity, solid–liquid extraction

## Abstract

The antioxidant potential of plant extracts is frequently evaluated using total phenolic content (TPC), although TPC alone provides limited information about the qualitative composition of phenolic compounds. This study investigated whether extraction conditions influence the antioxidant activity of *Lycium barbarum* extracts through selective remodeling of their phenolic fingerprint rather than solely through differences in TPC. A total of 160 extraction variants were prepared from conventionally dried and freeze-dried berries using four different solvent systems (water, 70% ethanol, 50% tert-butanol and 50% 1,4-dioxane) at four extraction temperatures (25, 37, 55 and 70 °C) and five extraction times (0.5, 2, 24, 48 and 72 h). Liquid extract recovery, TPC and antioxidant activity were determined using spectrophotometric assays, while selected food-relevant water and ethanol extracts were further characterized by high-performance liquid chromatography with diode-array detection (HPLC-DAD). Extraction conditions markedly affected liquid extract recovery, TPC and antioxidant activity; however, the highest TPC values did not consistently correspond to the strongest antioxidant responses. Correlations between TPC and antioxidant activity were moderate overall and varied with extraction conditions, indicating that TPC alone did not fully explain antioxidant performance. Chromatographic analysis showed that extraction selectively altered the abundance of individual phenolic classes and compounds, generating distinct phenolic fingerprints associated with different antioxidant responses. Together, these findings demonstrate that extraction conditions shape the antioxidant properties of *L. barbarum* extracts through both quantitative and qualitative changes in the recovered phenolic fraction, providing a basis for selecting extraction conditions and standardizing phenolic-rich ingredients for food and nutraceutical applications.

## 1. Introduction

Plant-derived bioactive compounds play an important role in the development of functional foods and nutraceuticals because of their potential contribution to health promotion and the prevention of disorders associated with oxidative stress [1,2]. Among these compounds, phenolic constituents are of particular interest owing to their antioxidant, anti-inflammatory, antimicrobial and metabolic properties [1,3]. However, the functional properties of plant extracts depend not only on the total amount of phenolic compounds recovered but also on their qualitative composition, which can be substantially modified by extraction and processing conditions [1,3].

*Lycium barbarum* (goji berry) is a widely recognized functional food used in traditional Asian medicine and increasingly incorporated into foods, beverages and dietary supplements [4,5]. Its fruits contain polysaccharides, carotenoids, phenolic acids, flavonoids, vitamins, amino acids and organic acids [3,4,5]. Phenolic compounds represent an important component of this phytochemical profile and contribute to the antioxidant properties of goji berries [4,5]. Previous studies demonstrate considerable quantitative variability in this fraction. For example, total phenolic content (TPC) reported for *L. barbarum* extracts has ranged from 14.13 mg gallic acid equivalents (GAE)/g in an aqueous fraction to 109.72 mg GAE/g in an ethyl acetate fraction [6], while pressurized ethanol–water extraction yielded up to 74.84 mg GAE/g [7]. In samples of different geographical origin extracted with water or aqueous ethanol, substantially lower values of approximately 3.31–23.49 mg GAE/g have also been reported [8]. Antioxidant capacity of *L. barbarum* extracts has also been reported to vary considerably with extraction solvent and processing method [6,9,10]. These differences illustrate that the phenolic characteristics of goji extracts are strongly influenced not only by the plant material itself but also by the extraction system applied.

The chemical composition of a plant extract is governed by interactions among the physicochemical properties of the target compounds, the plant matrix and the extraction system [1,11,12]. Solvent polarity, extraction temperature and extraction time determine compound solubility, diffusion and stability, whereas pretreatments such as drying can modify cellular structure and compound accessibility [1,11,12,13]. Studies specifically involving goji berries have shown that solvent selection may markedly alter phenolic recovery, while drying treatments can change both TPC and the recovery of individual phytochemicals [3,14,15,16]. Beyond phenolic content, extraction temperature and solvent composition have also been shown to shape antioxidant capacity in goji extracts: a response-surface optimization of pressurized liquid extraction identified 180 °C and 86% ethanol as the conditions maximizing radical-scavenging antioxidant activity [7]. Extraction temperature and time have similarly been optimized for goji antioxidant recovery using alternative technologies such as response-surface methodology and ohmic heating-assisted extraction [17,18], while solvent selection has also been shown to significantly affect bioactive compound recovery in dried goji berries [15,16].

Chromatographic studies have additionally identified substantial variability in compounds such as rutin, chlorogenic acid, catechin and epicatechin depending on cultivar, geographical origin, ripening stage and processing conditions [5,14,19]. These findings indicate that extraction should be regarded as a selective process capable of modifying the phenolic fingerprint rather than simply increasing or decreasing total phenolic recovery.

Despite this compositional complexity, extraction performance is still frequently assessed using extraction yield, TPC or antioxidant assays such as 2,2-diphenyl-1-picrylhydrazyl (DPPH) and 2,2′-azino-bis(3-ethylbenzothiazoline-6-sulfonic acid) (ABTS) [1,6]. These methods are rapid and useful for screening large sample sets, but they provide only indirect information about the qualitative composition of the extracts [1,20]. The Folin–Ciocalteu assay reflects overall reducing capacity of a sample rather than the selective quantification of individual phenolic constituents; non-phenolic reducing compounds can contribute to the measured response [21,22]. DPPH and ABTS assays differ in reaction environment, kinetics and sensitivity toward antioxidants with different structural and physicochemical properties [23,24]. Consequently, extracts with comparable TPC values may display substantially different antioxidant responses because of differences in their individual compounds, phenolic subclass distribution and potentially interacting constituents [23,24].

Previous studies have characterized the phenolic composition of *L. barbarum* under selected extraction or processing conditions [5,7,14,25]. However, the combined effects of drying pretreatment, solvent type, temperature and extraction time on composition–activity relationships remain insufficiently resolved. In particular, it is still unclear to what extent differences in antioxidant activity across a broad range of extraction conditions can be explained by total phenolic content alone, or whether they instead reflect extraction-induced changes in the qualitative phenolic fingerprint. Relatively few studies have integrated process recovery, TPC, more than one antioxidant assay and chromatographic profiling within the same experimental framework to address this question [14,25].

Therefore, the present study aimed to determine whether the antioxidant activity of *L. barbarum* extracts is governed primarily by extraction-induced changes in phenolic composition rather than by total phenolic content alone. Unlike previous investigations, this study integrates process performance, spectrophotometric assays and targeted chromatographic profiling within a single experimental framework, providing novel mechanistic insight into the compositional basis of antioxidant variability in goji berry extracts.

## 2. Results and Discussion

### 2.1. Liquid Extract Recovery (LER)

Across the 160 extraction variants, LER ranged from 6.84% (dried berries, water, 37 °C, 0.5 h) to 83.74% (freeze-dried berries, tert-butanol, 37 °C, 0.5 h). In this study, LER was treated as an operational technological parameter describing the proportion of the initial extraction mixture recovered as clarified liquid after filtration and centrifugation, rather than as a conventional dry extract yield or a direct measure of bioactive-compound extraction efficiency.

Factorial analysis of variance (ANOVA) revealed that solvent type was the dominant factor affecting filtrate recovery, followed by extraction time and temperature (*p* < 0.001; Table 1).

In contrast, the drying pretreatment of the berries had no significant effect on liquid extract recovery (*p* = 0.470), with dried and freeze-dried samples showing nearly identical mean recoveries (57.06 ± 18.57% and 56.18 ± 18.73%, respectively). Nevertheless, drying method interacted significantly with both solvent and time (*p* < 0.001; Table 1), indicating that its influence on liquid extract recovery was not uniform but depended on the extraction conditions applied.

A factorial general linear model (GLM) explained 88.7% of the variability in liquid extract recovery (R^2^ = 0.887).

Among the tested solvents, tert-butanol and 1,4-dioxane consistently produced the highest recoveries, followed by 70% aqueous ethanol, while water gave markedly lower and more variable recovery (Table 2). The lower recovery obtained for water most likely reflects stronger solvent retention within the hydrated plant matrix and less efficient phase separation, rather than lower extraction efficiency alone.

Previous work on *L. barbarum* likewise identified solvent composition as a major determinant of extraction performance [7,26,27].

The significant solvent × time interaction (*p* < 0.001; Table 1) was driven mainly by the time-dependent behavior of aqueous extracts. At 37 °C, for example, water LER increased from 7.4% after 0.5 h to 69.4% after 72 h, whereas the organic solvent systems achieved comparatively high recovery already at short extraction times. This behavior is compatible with time-dependent solvent penetration, matrix hydration and mass-transfer processes during solid–liquid extraction [28].

Overall, these results support interpretation of LER as a complementary process-recovery metric that should be considered alongside TPC, antioxidant activity and chromatographic profiling, rather than as a measure of selective phenolic extraction [29,30].

### 2.2. Total Phenolic Content (TPC) of Extracts

The total phenolic content (TPC) of *L. barbarum* extracts was significantly affected by the extraction conditions, ranging from 23.82 to 75.74 mg GAE/g dry weight (DW) across all extraction variants. Factorial analysis of variance showed that solvent type (F = 60.40) and extraction temperature (F = 61.98) contributed comparably as the strongest factors (Table 3), together with significant effects of extraction time and drying pretreatment (all *p* < 0.001) and significant interactions among these variables. A factorial general linear model explained 87.5% of the variability in TPC (R^2^ = 0.875), confirming that phenolic recovery could not be optimized by adjusting a single extraction parameter but required consideration of the combined effect of process variables, in accordance with literature data [26,31,32].

Among the solvents tested, 50% aqueous 1,4-dioxane resulted in the highest TPC (55.39 ± 10.12 mg GAE/g DW), followed by 50% aqueous tert-butanol (49.94 ± 9.53 mg GAE/g DW), 70% aqueous ethanol (46.21 ± 10.30 mg GAE/g DW), and water, which yielded the lowest phenolic concentration (41.45 ± 9.38 mg GAE/g DW)—approximately 34% lower than 1,4-dioxane. A comparable, even more pronounced solvent effect has been reported for organic-solvent fractionation of *L. barbarum*, with TPC ranging from 14.13 mg GAE/g (water fraction) to 109.72 mg GAE/g (ethyl acetate fraction) [6], while pressurized liquid extraction of goji berries reached up to 74.84 mg GAE/g under optimized ethanol–water conditions [7]—a value closely matching the maximum obtained here. These species-specific comparisons support the interpretation that *L. barbarum* phenolics are preferentially recovered by organic or intermediate-polarity solvents rather than water alone. The complete factorial pattern is shown in Figure 1.

Extraction temperature significantly influenced TPC, with the highest values at 55 °C, whereas a further increase to 70 °C reduced phenolic content. This pattern indicates that moderate heating enhanced mass transfer, solvent penetration and solute diffusion, while more intensive heating increasingly promoted oxidation, degradation or structural transformation of thermolabile phenolics. This balance depends strongly on extraction technology: under pressurized liquid extraction, goji berry TPC increased from 5.72 to 74.84 mg GAE/g between 50 °C and 180 °C (86% ethanol), showing that elevated pressure permits aggressive matrix disruption without solvent boiling [7], whereas atmospheric-pressure optimization identified a comparatively moderate temperature optimum of 45 °C and 162 min, yielding 1338.8 mg GAE/100 g (13.39 mg GAE/g) [17]. Thermal-stability studies on *L. barbarum* showed that phenolic degradation became markedly more pronounced between 60 and 70 °C, reflected in a higher temperature coefficient (Q10 = 2.17) over this range than over 50–60 °C (Q10 = 1.53) [33]; a similarly protective effect of moderate heating (65 °C) over more intensive treatment has been reported in raspberries, boysenberries, redcurrants and blackcurrants [34]. Collectively, these results support the interpretation that the optimum temperature reflects a compromise between enhanced mass transfer and preservation of thermolabile phenolics, rather than a simple temperature-dependent increase in extraction efficiency.

Extraction time also significantly affected TPC. The rapid TPC increase observed during the initial extraction stage is consistent with the fast release of readily accessible phenolic compounds from disrupted plant tissues, whereas the subsequent plateau and, under some conditions, slight decline indicate progressive attainment of extraction equilibrium, with prolonged extraction potentially allowing oxidative or thermal degradation to offset further phenolic release, as shown for black currants [28] and for *Cayratia trifolia* berries [35]. A finite extraction window has also been demonstrated quantitatively for *L. barbarum*: response-surface optimization under atmospheric pressure identified an optimum of approximately 162 min at 45 °C, yielding 1338.8 mg GAE/100 g [17]—a much shorter window than the 24–48 h observed here, reflecting differences in extraction system and operating conditions, though both datasets show that phenolic recovery eventually reaches a plateau. Extraction time should therefore be regarded as an optimization rather than a maximization parameter, because extending the process beyond the optimal duration may increasingly allow equilibrium and degradation processes to offset additional phenolic release.

Drying pretreatment of goji berries significantly affected TPC, although its effect was weaker than that of solvent, temperature and extraction time. Conventionally dried berries generally exhibited slightly higher TPC than freeze-dried material; however, significant interactions with the remaining extraction variables indicate that this effect was condition-dependent rather than uniform (Figure 1). Quantitative comparisons in the literature likewise show that no drying method is universally superior. Spray-dried goji berry powder has been reported to exhibit higher TPC than freeze-dried powder when extracted with water, ethanol or methanol at comparable concentrations [15], whereas studies using freeze-drying, vacuum drying and hot-air drying have often reported better retention of phenolics under low-temperature or oxygen-limited conditions in other fruit matrices [36,37,38] and in goji specifically [16]. These apparently contradictory findings are consistent with the HPLC results of the present study: freeze-drying of goji berries increased mean flavan-3-ols to 55.53 mg/100 g DW in water extracts compared with 26.43 mg/100 g DW in extracts of conventionally dried ones. Moreover, conventionally dried berry extracts contained more flavonols (55.67 vs. 28.24 mg/100 g DW), mainly because of higher rutin concentrations. Thus, the slightly higher TPC of conventionally dried berry extracts should not be interpreted as universally superior phenolic preservation. Rather, drying pretreatment probably resulted in a redistribution of the recovered phenolic fraction among subclasses, demonstrating that total phenolic quantity and phenolic composition must be evaluated together.

To determine whether improved liquid extract recovery translates into increased phenolic recovery, the relationship between LER and TPC of all extracts tested was examined. A statistically significant but only moderate positive correlation was observed (Pearson’s *r* = 0.41, *p* < 0.001), indicating that higher liquid recovery did not directly determine phenolic concentration. The divergence was also evident from the ranges of the two variables: LER changed more than twelve-fold (6.84–83.74%), whereas TPC changed only about three-fold (23.82–75.74 mg GAE/g DW). These results are in agreement with extraction studies of bean extract showing that conditions maximizing overall extraction yield did not necessarily maximize TPC value, because highly polar solvents favored bulk recovery over selective recovery of phenolic constituents [26]. Accordingly, LER and TPC describe complementary rather than interchangeable aspects of extraction performance: LER reflects process and phase-separation efficiency, whereas TPC reflects selectivity toward reducing phenolic constituents.

For the food-grade solvent tested, 70% ethanol, the highest TPC was 68.38 mg GAE/g DW (conventionally dried berries at 55 °C for 48 h)—approximately 8.6% lower than the goji berry extract TPC reported by Tripodo et al. [7] under optimized pressurized liquid extraction, suggesting that carefully selected atmospheric-pressure conditions with a comparable food-grade solvent can approach the phenolic recovery achieved by more intensive, pressurized technology. In our previous study, a water extract from a different *L. barbarum* harvest batch at 70 °C for 72 h had a TPC of 63.6 mg GAE/g DW [39], slightly below the water-extract maximum obtained here (66.74 mg GAE/g DW, dried material at 55 °C for 48 h). By contrast, goji berry samples of different geographic origin extracted with water and 50% ethanol contained only 3.31–23.49 mg GAE/g [8], illustrating that such variation among studies likely reflects differences in plant material, harvest season, and extraction technology rather than solvent choice alone.

TPC determined by the Folin–Ciocalteu assay should be interpreted as an estimate of the overall reducing capacity expressed as gallic acid equivalents rather than an absolute measure of phenolic concentration [40,41,42]. Nevertheless, TPC is generally considered a useful proxy for the reducing capacity of plant extracts and frequently correlates with antioxidant activity, although the strength of this relationship depends on the composition of the phenolic fraction [31,41,42,43,44].

### 2.3. DPPH Radical Scavenging Activity Reflects Phenolic Recovery

The antioxidant activity of *L. barbarum* extracts, determined by the DPPH assay and expressed as Trolox equivalent antioxidant capacity (TEAC), ranged from 50.52 to 239.57 µmol Trolox equivalent (TE)/g DW—a nearly five-fold variation confirming that extraction conditions substantially affected the recovery of radical-scavenging constituents. A comparable multi-parameter optimization has been reported for goji berry using ohmic heating-assisted extraction, which identified temperature and time, together with electrical field strength, as joint determinants of DPPH activity, with an optimum at 60 °C and 60 min [18].

Factorial ANOVA showed that solvent type, extraction temperature, extraction time and drying pretreatment all significantly affected DPPH activity (*p* < 0.001), with extraction temperature exerting the strongest effect—indicating that recovery of antioxidant-active constituents was particularly sensitive to thermal conditions, and that the temperature maximizing DPPH activity did not simply correspond to the most intensive treatment. Among the solvents tested, 50% 1,4-dioxane produced the highest mean extract DPPH activity (147.88 ± 51.74 µmol TE/g DW), followed by 50% tert-butanol (137.58 ± 48.93 µmol TE/g DW) and 70% ethanol (133.09 ± 50.67 µmol TE/g DW), whereas water yielded the lowest activity (114.55 ± 41.38 µmol TE/g DW). Quantitative solvent-dependent differences have also been reported for goji extracts: Vulić et al. [9] found DPPH values ranging from 26.64 µmol TE/g for a hexane extract to 62.15 µmol TE/g for a methanolic extract, while Benchennouf et al. [6] reported the lowest activity in a water fraction (14.13 mg GAE/g) and the highest in an ethyl acetate fraction (109.72 mg GAE/g). Although these studies used different extraction and reporting systems, they consistently indicate that aqueous extraction recovers a less antioxidant-active fraction than solvents of intermediate polarity. The complete factorial pattern is shown in Figure 2.

The observed DPPH range was higher than many values reported for untreated goji fruit extracts but remained within the broader range obtained after intensive processing. Methanolic and hexane goji extracts reported by Vulić et al. [9] ranged from 26.64 to 62.15 µmol TE/g, while Zhang et al. [45] reported 35.88–85.46 µmol TE/g fresh weight across eight Chinese goji samples—not directly comparable on a dry-weight basis. By contrast, fermented *L. barbarum* juice reached 343.67 µmol TE/g dry weight [46], showing that processing can increase DPPH activity well beyond the upper value observed here. Collectively, these comparisons place the present extracts above many conventionally prepared goji fruit extracts but below strongly modified fermented preparations.

TPC and DPPH were positively but only moderately correlated (*r* = 0.611, *p* < 0.001, *r^2^* ≈ 0.373)—weaker than the R^2^ = 0.75 reported by Aryal et al. [47] for wild vegetable extracts and the R^2^ = 0.771 reported by Khiya et al. [48] for Salvia officinalis extracts. This lower explanatory power indicates that total phenolic content alone did not capture the effects of phenolic composition, drying pretreatment and extraction selectivity on DPPH activity.

DPPH activity showed a non-linear temperature response, reaching its highest levels around 55 °C and declining at 70 °C, indicating that moderate heating enhanced recovery of active antioxidants, whereas further increase reduced their recovery and/or stability. A comparable moderate-temperature optimum was reported for organically grown goji berries by Pedro et al. [17], who identified 45 °C, 162 min and a 1:30 solid-to-solvent ratio as optimal, yielding 2.81 mmol TE/100 g by the DPPH assay—a lower absolute value reflecting differences in raw material, solvent system and extraction design, though the temperature response similarly indicates that increasing temperature does not indefinitely improve antioxidant recovery [18].

Extraction time produced a similar finite optimum: DPPH activity increased during the initial stages, was highest mainly between 24 and 48 h, and declined after 72 h, consistent with progressive release of antioxidant compounds followed by extraction equilibrium, while prolonged exposure may allow degradation of labile constituents to offset further recovery. Pedro et al. [17] identified an optimum of 162 min for atmospheric-pressure extraction of organic goji material, whereas accelerated solvent extraction of black goji (*Lycium ruthenicum*) achieved its optimum at only 13 min [49]—not directly comparable with the 24–48 h optimum observed here given the substantially different extraction technologies, though all three datasets confirm that extraction time is an optimization rather than a maximization parameter.

Freeze-dried berries produced higher mean DPPH activity than conventionally dried material (142.24 ± 50.82 vs. 124.31 ± 46.54 µmol TE/g DW, +14.4%), despite their slightly lower mean TPC. A comparable tendency was observed in another phenolic-rich plant matrix, *Triadica sebifera* leaves, for which Fan et al. [50] reported extract DPPH half-maximal inhibitory concentration (IC50) values of 135.5 µg/mL after freeze-drying and 138.2 µg/mL after vacuum drying, versus 159.1 µg/mL after hot-air drying—a lower IC50 indicates stronger scavenging activity, i.e., freeze-drying improved DPPH performance by approximately 15% relative to hot-air drying. This divergence indicates drying pretreatment altered the composition and radical-scavenging potency of the recovered fraction rather than simply changing total phenolic abundance.

The HPLC results in Section 2.5 are consistent with this interpretation: freeze-dried extracts were richer in flavan-3-ols, whereas conventionally dried extracts contained more flavonols.

Overall, DPPH activity was strongly shaped by extraction temperature and further modified by solvent, time and drying pretreatment.

### 2.4. ABTS Assay Confirms Trends Observed for DPPH

ABTS activity ranged from 2.67 to 159.63 µmol TE/g DW (mean 79.64 ± 27.63 µmol TE/g DW). Temperature produced the clearest response, with the highest mean activity at 55 °C (104.92 µmol TE/g DW) compared with 68.35, 76.89 and 68.41 µmol TE/g DW at 25, 37 and 70 °C, respectively, while activity increased up to 48 h (100.76 µmol TE/g DW) and declined at 72 h (77.53 µmol TE/g DW). Mean values also differed among solvents, from 67.24 ± 33.50 µmol TE/g DW for 50% 1,4-dioxane to 90.14 ± 25.33 µmol TE/g DW for 70% ethanol, and between drying treatments, with conventionally dried extracts slightly higher than freeze-dried ones (83.48 ± 31.05 vs. 75.81 ± 23.30 µmol TE/g DW)—confirming that ABTS response was extraction-dependent but not directly proportional to TPC. The complete factorial pattern is shown in Figure 3.

Aqueous extracts of Greek-cultivated goji berries showed ABTS IC50 values of 0.39–1.10 mg/mL and DPPH IC50 values of 1.29–3.00 mg/mL [10], confirming that the same extracts can respond differently in the two assays. On a TEAC basis, dried goji fruit extracts have been reported at 58.3 µmol TE/g [51] and 53.92–64.38 µmol TE/g for red-cultivar samples [52], whereas dried and freeze-dried aqueous and ethanolic extracts ranged from approximately 39.8 to 46.5 µmol TE/g [8]. Fermented *L. barbarum* juice reached 229.89 µmol TE/g dry weight [46], substantially exceeding the maximum of 159.63 µmol TE/g DW observed here. Thus, the current ABTS range is higher than many conventionally processed dried fruit extracts, but lower than that achieved after fermentation, highlighting the strong influence of processing route on antioxidant response.

Across the complete dataset, TPC correlated moderately with DPPH (r = 0.611, *p* < 0.001) and more weakly with ABTS (r = 0.393, *p* < 0.001). DPPH and ABTS were also only moderately correlated (r = 0.439, *p* < 0.001; r^2^ ≈ 0.193), whereas LER was unrelated to ABTS activity (r = −0.005, *p* = 0.946) (Table 4). These relationships show that the two radical assays captured overlapping but non-identical properties of the extracts, and that liquid-phase recovery did not predict ABTS activity. Notably, this relationship was solvent- and condition-dependent: for water extracts specifically, TPC correlated more strongly with both DPPH and ABTS at 25–55 °C for 24–72 h (r = 0.618 and r = 0.630, respectively, both *p* < 0.01) than under harsher conditions (70 °C or extraction times of 0.5–2 h), where the correlation weakened or lost significance.

The complete pairwise correlation structure among LER, TPC, DPPH, and ABTS is summarized in Table 4 and Figure 4.

Stratification by drying pretreatment further showed that these relationships were composition-dependent (Table 5).

Most notably, the relationship between TPC and ABTS differed sharply according to drying pretreatment. For conventionally dried berries, TPC and ABTS were moderately correlated (r = 0.590, *p* < 0.001), whereas for freeze-dried berries the relationship was negligible and non-significant (r = 0.114, *p* = 0.316). The TPC–DPPH relationship was more stable, though still weaker after freeze-drying (r = 0.742 vs. 0.581). Similarly, the LER–DPPH correlation decreased from r = 0.511 to r = 0.229, while LER–ABTS remained non-significant in both groups (r = 0.125 and −0.186). This drying-dependent decoupling is consistent with the HPLC results in Section 2.5, where freeze-dried extracts were enriched in flavan-3-ols and conventionally dried extracts in flavonols—subclasses that differ in their reactivity toward ABTS and DPPH radicals—so that the predictive value of TPC varied with phenolic composition rather than remaining fixed across pretreatments [53].

The moderate global correlations indicate that total phenolic content cannot be regarded as a universal predictor of antioxidant activity. This contrasts with datasets in which the two assays track each other closely: Floegel et al. [54], analyzing 50 antioxidant-rich foods, reported a Spearman correlation of ρ = 0.949 between ABTS and DPPH—far higher than the r = 0.439 observed here, reflecting the deliberately broad variation in solvent, drying pretreatment, temperature and extraction time in the present design. For example, a dried water extract (70 °C, 24 h) and a freeze-dried ethanol extract (37 °C, 48 h), both with identical TPC (44.12 mg GAE/g DW), showed markedly different DPPH responses (72.35 vs. 235.80 µmol TE/g DW), demonstrating that the relationship between total reducing capacity and radical-scavenging activity was extraction-dependent.

This interpretation is supported by the distinct chemical selectivity of the DPPH and ABTS assays. Although both are predominantly single-electron-transfer methods, ABTS can be applied in both aqueous and organic environments, whereas DPPH is more constrained by organic-solvent conditions. Platzer et al. [53], comparing 24 individual phenolic compounds, showed that several dihydrochalcones and flavanones reacted with ABTS but showed little or no DPPH response, and that the ranking of reactive phenolic acids also differed between assays. Such compound-specific behavior explains why ABTS and DPPH can diverge even for the same extract; accordingly, the moderate agreement observed here should be interpreted as complementary analytical information about phenolic composition and reactivity.

Taken together, the TPC, DPPH and ABTS results show that extraction conditions altered antioxidant response in an assay-dependent manner: TPC–ABTS correlation was weak overall and absent for freeze-dried extracts, while DPPH and ABTS shared less than one-fifth of their variance. These quantitative differences show that antioxidant properties of *L. barbarum* extracts cannot be inferred reliably from TPC, thereby providing the rationale for targeted HPLC-DAD profiling of selected water and ethanol extracts to examine whether differences in phenolic composition accompanied the observed functional divergence.

### 2.5. HPLC-DAD Profiling Provides Compositional Context for Antioxidant Responses

The results presented in Section 2.1, Section 2.2, Section 2.3 and Section 2.4 showed that liquid extract recovery, total phenolic content (TPC), and antioxidant activity describe complementary but non-equivalent aspects of the extraction process. Across the complete dataset, TPC correlated moderately with DPPH (r = 0.611, *p* < 0.001) and more weakly with ABTS (r = 0.393, *p* < 0.001), while DPPH and ABTS were only moderately correlated (r = 0.439, *p* < 0.001). These associations were substantially weaker than those reported by Dudonné et al. [55] for 30 aqueous plant extracts, where Folin–Ciocalteu assay values correlated with DPPH at R = 0.939 and with ABTS at R = 0.966, indicating that the broad factorial variation applied here generated extracts whose antioxidant behavior could not be explained by phenolic quantity alone. HPLC-DAD profiling of a targeted subset was therefore used to examine whether the observed functional differences were accompanied by changes in the distribution of quantified phenolic compounds.

Eight extracts were selected to represent both berry pretreatments (conventionally dried and freeze-dried), both food-relevant solvents (water and 70% ethanol), and two contrasting technological extraction strategies: 55 °C for 48 h and 70 °C for 30 min. The former produced high TPC and antioxidant responses among the water and ethanol systems, whereas the latter represented a short, infusion-like preparation. Water and ethanol were selected because they are directly relevant to food and nutraceutical applications; 1,4-dioxane and tert-butanol were used only as comparative extraction media in the broader factorial experiment.

Importantly, temperature and time differed simultaneously between the two HPLC strategies: their comparison therefore represents two complete technological extraction strategies and cannot be used to infer an isolated effect of temperature or extraction time—a distinction reinforced by the literature, where moderate-temperature optima depend strongly on extraction technology (Pedro et al. [17]: 45 °C, 162 min, 70% ethanol–water under atmospheric pressure; Tripodo et al. [7]: 180 °C, 86% ethanol under pressurized liquid extraction). The HPLC subset was selected for targeted compositional interpretation and was not treated as a separate full-factorial experiment.

HPLC analysis showed that the selected extracts contained compounds assigned to the principal phenolic groups quantified in this study, while their relative distributions varied among extraction conditions. The resulting compositional patterns are presented in Figure 5.

The chromatographic profiles indicated marked differences in the relative distribution of the quantified phenolic groups among the selected extracts, with the most pronounced shifts in extract composition associated with berry drying pretreatment, while solvent selection primarily modified the relative contributions of flavonols and hydroxycinnamic acids. Because the eight-extract HPLC subset was evaluated descriptively, these patterns are interpreted as compositional trends rather than statistically demonstrated factor effects—indicating that the investigated conditions altered the quantitative distribution of phenolic subclasses rather than their qualitative occurrence. A quantitative summary of these descriptive differences associated with berry pretreatment and extraction solvent is presented in Table 6.

Descriptively, the clearest contrast associated with berry pretreatment concerned the balance between flavan-3-ols and flavonols. Extracts from freeze-dried berries contained an average of 55.53 mg/100 g DW of flavan-3-ols, compared with 26.43 mg/100 g DW for conventionally dried material. Epicatechin accounted for most of this difference, ranging from 38.84 to 53.35 mg/100 g DW in extracts from freeze-dried berries and from 14.33 to 26.60 mg/100 g DW in extracts from conventionally dried berries. Conversely, the mean flavonol concentration was 55.67 mg/100 g DW for conventionally dried material and 28.22 mg/100 g DW for freeze-dried material. Apigenin, which was detected only at trace levels (0.03–0.05 mg/100 g DW in four extracts), was classified separately as a flavone and was not included in the flavonol totals. Thus, the selected extracts suggest that berry pretreatment was associated with a redistribution among phenolic groups rather than a uniform increase or decrease in all quantified constituents. Mocan et al. [56] reported rutin concentrations of approximately 200–400 µg/g (20–40 mg/100 g) across European goji cultivars—within the same order of magnitude as, though generally below, the 22.51–60.47 mg/100 g DW range observed here, with direct comparison limited by differences in plant material and analytical procedures.

Solvent-related patterns were also compound-class-dependent. Across the selected extracts, ethanol was associated with a higher mean flavonol concentration than water (45.96 vs. 37.92 mg/100 g DW), largely reflecting rutin, whereas aqueous extracts showed a higher mean concentration of hydroxycinnamic acids (16.31 vs. 11.34 mg/100 g DW). Mean flavan-3-ol concentrations were similar in ethanol and water extracts (40.87 vs. 41.10 mg/100 g DW). These descriptive data therefore suggest solvent-dependent selectivity rather than a uniform solvent effect across all phenolic groups. Rutin, chlorogenic acid, catechin, and epicatechin have also been reported as recurrent phenolic constituents of *L. barbarum* [56,57]. A direct comparison of the two technological extraction strategies is presented in Table 7.

Several factors may contribute to this discrepancy. The Folin–Ciocalteu assay reflects overall reducing capacity and is not specific to phenolic compounds, whereas the targeted HPLC-DAD method quantifies only the compounds included in the analytical panel. Unquantified reducing or antioxidant-active constituents, including ascorbic acid and other non-phenolic reducing species, as well as water-soluble polysaccharide-associated components or processing-derived reaction products, could therefore contribute to the spectrophotometric responses; these were not quantified here, so their contribution remains a plausible explanation rather than a demonstrated mechanism. Dudonné et al. [55] observed very strong correlations between Folin–Ciocalteu assay values and DPPH (R = 0.939) and ABTS (R = 0.966) across 30 aqueous plant extracts, but the much weaker relationships in the present factorial dataset show that such agreement is not universal. In addition, individual phenolic compounds differ in their reactivity toward DPPH and ABTS: Platzer et al. [53], testing 24 individual phenolic compounds, showed that several dihydrochalcones and flavanones reacted with ABTS but little or not at all with DPPH, and that phenolic acids were ranked differently by the two assays. The spectrophotometric and chromatographic measurements should therefore be interpreted as complementary rather than interchangeable.

The correlation analysis supports the same cautious interpretation. TPC shared approximately 37.3% of its variance with DPPH and 15.4% with ABTS, while DPPH and ABTS shared approximately 19.3%; the TPC-linked correlations were markedly lower than the R = 0.939 and 0.966 reported by Dudonné et al. [55] for a more compositionally homogeneous set of aqueous extracts, consistent with the deliberate variation in solvent, pretreatment, temperature and time applied here. Together with the descriptive HPLC-DAD profiles, the results support an extraction-dependent relationship between phenolic quantity, phenolic composition, and assay response, without establishing a causal contribution of any individual compound or phenolic group.

## 3. Materials and Methods

### 3.1. Plant Material and Experimental Design

Dried *L. barbarum* (goji berry) fruits originating from the Ningxia Autonomous Region (China) were supplied by NingXia Senmiao Technology and Development Co., Ltd. (Chengdu, China). Two commercially available forms of the plant material were investigated: conventionally air-dried and freeze-dried berries. Upon receipt, the samples were stored in airtight containers in a dry, dark environment at room temperature until analysis. Before extraction, the fruits were ground to a homogeneous powder using a laboratory mill (06-HC300, Chemland, Stargard, Poland). The harvest year of the commercially obtained fruits was not provided by the supplier. Results reported on a dry-weight (DW) basis were calculated directly from the mass of the dried (or freeze-dried) berry material as weighed immediately before extraction, rather than from an independently determined, moisture-corrected dry-matter value; a separate moisture-content determination was not performed. This operational DW basis was adopted because the plant material was used in its commercially supplied dried form without further processing, so that weighing the dried starting material immediately before extraction provided a practical and reproducible normalization basis applicable uniformly across the full factorial design, and because expressing results per gram of dried starting material follows the convention commonly used in extraction and antioxidant-activity studies of dried plant materials, allowing for comparison with previously published data for *L. barbarum*. As a limitation, because no residual moisture determination was performed, the commercially dried material may retain a small residual moisture content, so values expressed on this operational DW basis may differ slightly from values calculated on a fully moisture-corrected dry-matter basis. This operational basis was applied uniformly across all extraction variants, so comparisons among conditions within the present study remain valid.

A full-factorial experimental design was used to evaluate the effects of drying pretreatment, extraction solvent, temperature and time on liquid extract recovery, phenolic recovery and antioxidant activity. Both plant materials were extracted using four solvents differing in polarity: distilled water, 70% (*v*/*v*) aqueous ethanol, 50% (*v*/*v*) aqueous tert-butanol and 50% (*v*/*v*) aqueous 1,4-dioxane. Extractions were performed at 25, 37, 55 or 70 °C for 0.5, 2, 24, 48 or 72 h, giving 160 extraction variants (2 drying pretreatments × 4 solvents × 4 temperatures × 5 extraction times). Each extraction was prepared independently in triplicate.

All extracts were evaluated for liquid extract recovery (LER), total phenolic content (TPC), and antioxidant activity by the DPPH and ABTS assays. HPLC-DAD profiling was intentionally restricted to a targeted subset of eight food-relevant extracts prepared with water or 70% ethanol. The subset represented both drying pretreatments and two contrasting technological extraction strategies: 55 °C for 48 h, which yielded high antioxidant responses among the food-grade solvent systems, and 70 °C for 30 min, selected as a short, infusion-like extraction scenario. Because extraction temperature and time differed simultaneously between these two strategies, their comparison was used to contrast complete extraction conditions and not to infer an isolated effect of either temperature or extraction time. The HPLC subset was therefore used for targeted compositional interpretation and was not treated as a separate full-factorial experiment.

### 3.2. Chemicals and Reagents

Unless otherwise stated, chemicals, extraction solvents and analytical standards used in this study were of analytical or HPLC grade and were purchased from Sigma-Aldrich (Merck KGaA, Darmstadt, Germany). DPPH, ABTS, Folin–Ciocalteu reagent, gallic acid, and Trolox were obtained from the same supplier. HPLC-grade acetonitrile, methanol, formic acid, and ultrapure water were used for preparation of the mobile phases and chromatographic analyses.

The aqueous tert-butanol and 1,4-dioxane systems were included solely to broaden the physicochemical range of extraction media for comparative and mechanistic purposes; they are not proposed as solvent systems for food or nutraceutical applications. Accordingly, HPLC-DAD profiling was restricted to extracts prepared with water and 70% ethanol.

All reference standards used for HPLC-DAD analysis were purchased from Sigma-Aldrich (St. Louis, MO, USA) and were of HPLC-grade purity (>95%). Catechin, epicatechin, apigenin, myricetin, gallic acid, vanillic acid, 3,4-dihydroxybenzoic acid (protocatechuic acid), 4-hydroxybenzoic acid, caffeic acid, ferulic acid, *p*-coumaric acid, and chlorogenic acid were identified and quantified using the corresponding authentic reference standards. Quercetin and kaempferol were used as calibration standards for the semi-quantitative determination of their glycoside derivatives, as described in Section 3.8.

### 3.3. Preparation of Extracts

Ground *L. barbarum* fruits (6.0 g) were combined with 60 mL of the appropriate extraction solvent in sealed glass vessels, corresponding to a solid-to-solvent ratio of 1:10 (*w*/*v*). The four extraction systems were distilled water, 70% (*v*/*v*) aqueous ethanol, 50% (*v*/*v*) aqueous tert-butanol and 50% (*v*/*v*) aqueous 1,4-dioxane.

Extractions were carried out in a thermostatically controlled water bath under continuous agitation at four temperatures (25, 37, 55 and 70 °C) for 0.5, 2, 24, 48 or 72 h.

After extraction, the suspensions were filtered through Whatman No. 1 filter paper and centrifuged at 4500 rpm for 10 min using an Eppendorf 5804 R centrifuge (Eppendorf AG, Hamburg, Germany). The clarified liquid phase was collected and analyzed immediately after centrifugation.

For HPLC-DAD analysis, the selected water and ethanol extracts were additionally passed through 0.45 µm polytetrafluoroethylene (PTFE) syringe filters before injection. All extraction experiments were performed independently in triplicate.

### 3.4. Determination of Liquid Extract Recovery

Liquid extract recovery (LER) was determined gravimetrically for each extraction variant. Following the standardized filtration and centrifugation procedure described in Section 3.3, the mass of the recovered clarified liquid phase was recorded. LER was calculated according to Equation (1):

(1) $$LER\;(\%)\;=\;\frac{mass\;of\;filtrate\;recovered\;after\;extraction}{\;(mass\;of\;dried\;berries\;+\;mass\;of\;solvent)}\times 100$$

The denominator in Equation (1) represents the initial total mass introduced into the extraction system (dried berry material plus extraction solvent). LER was therefore defined as the fraction of the initial extraction mixture recovered as clarified liquid after solid–liquid separation. Liquid retained within the hydrated plant matrix or filter material and minor handling losses were not quantified separately and consequently contribute to this operational recovery parameter. Because the same filtration and centrifugation procedure was applied to all extraction variants, LER was used for relative comparison of phase-recovery behavior among extraction conditions. It should not be interpreted as conventional dry extract yield or as a direct measure of phenolic extraction efficiency. All determinations were performed in triplicate, and results are presented as mean ± standard deviation (SD).

### 3.5. Determination of Total Phenolic Content

Total phenolic content (TPC) was determined using the Folin–Ciocalteu colorimetric assay according to Singleton et al. [21], with minor modifications introduced by Skenderidis et al. [10].

Briefly, 100 µL of each extract was transferred to a 2 mL microcentrifuge tube and mixed with 100 µL of 0.2 N Folin–Ciocalteu reagent. After 3 min, 1.0 mL of 7% (*w*/*v*) sodium carbonate solution was added. The reaction mixtures were incubated for 60 min at room temperature in the dark, and absorbance was measured at 765 nm using a Shimadzu UV-1800 UV-Vis spectrophotometer (Shimadzu, Kyoto, Japan).

Quantification was performed by external calibration with gallic acid (0.02–0.08 mg mL^−1^; R^2^ = 0.9989). Samples requiring dilution to obtain an analytical response within the calibration range were appropriately diluted prior to measurement, and the corresponding dilution factors were included in the calculation of the final TPC values. Results were expressed as milligrams of gallic acid equivalents per gram of dry weight (mg GAE/g DW). All measurements were performed in triplicate, and results are presented as mean ± SD.

### 3.6. Determination of Antioxidant Activity by the DPPH Assay

Antioxidant activity was determined using the 2,2-diphenyl-1-picrylhydrazyl (DPPH) radical-scavenging assay according to Alzagameem et al. [58], with minor modifications.

Briefly, 100 µL of each extract was mixed with 3.90 mL of freshly prepared DPPH solution (6.0 × 10^−5^ mol L^−1^) prepared in a 90:10 (*v*/*v*) mixture of 1,4-dioxane and distilled water. The reaction mixtures were incubated for 30 min at room temperature in the dark, and absorbance was measured at 518 nm.

Quantification was performed by external calibration with Trolox (30–230 mg L^−1^; R^2^ = 0.9973). Antioxidant activity was expressed as Trolox equivalent antioxidant capacity (TEAC; µmol TE/g DW). All measurements were performed in triplicate, and results are presented as mean ± SD. Samples whose absorbance was outside the suitable measurement range were appropriately diluted prior to measurement, and the corresponding dilution factors were included in the calculation of the final antioxidant activity values.

### 3.7. Determination of Antioxidant Activity by the ABTS Assay

Antioxidant activity was also determined using the ABTS radical cation decolorization assay described by Re et al. [59], with minor modifications.

The ABTS radical cation (ABTS•+) was generated by reacting 7.0 mM ABTS with 2.45 mM potassium persulfate for 12 h at room temperature in the dark. Before analysis, the ABTS•+ solution was diluted with distilled water to an absorbance of 0.700 ± 0.005 at 734 nm.

For the assay, 2.50 mL of the diluted ABTS•+ solution was mixed with 250 µL of extract and incubated for 12 min at room temperature, after which the absorbance was measured at 734 nm.

Quantification was performed by external calibration with Trolox (15–40 mg L^−1^; R^2^ = 0.9972). Antioxidant activity was expressed as TEAC (µmol TE/g DW). All measurements were performed in triplicate, and results are presented as mean ± SD. Samples whose absorbance was outside the suitable measurement range were appropriately diluted prior to measurement, and the corresponding dilution factors were included in the calculation of the final antioxidant activity values.

### 3.8. HPLC-DAD Analysis of Phenolic Compounds

Phenolic compounds were analyzed using an Ultimate 3000 HPLC system (Thermo Fisher Scientific, Waltham, MA, USA) equipped with a diode-array detector (DAD). Separation was performed on an Accucore C18 analytical column (150 × 3.0 mm, 2.6 µm particle size) maintained at 30 °C. The injection volume was 2 µL and the flow rate was 0.35 mL min^−1^. The mobile phase consisted of 0.1% (*v*/*v*) formic acid in water (solvent A) and 0.1% (*v*/*v*) formic acid in acetonitrile (solvent B). Phenolic compounds were monitored at 280, 320, and 365 nm, corresponding to hydroxybenzoic acids/flavan-3-ols, hydroxycinnamic acids, and flavonols and flavones, respectively. Catechin, epicatechin, apigenin, myricetin, gallic acid, vanillic acid, protocatechuic acid, 4-hydroxybenzoic acid, caffeic acid, ferulic acid, *p*-coumaric acid, and chlorogenic acid were identified by comparison of retention times and UV-Vis spectra with the corresponding authentic reference standards and quantified using calibration curves prepared from the respective standards. Quercetin 3-O-galactoside, quercetin 3-O-glucoside, quercetin dihexoside, and quercetin 3-O-rutinoside (rutin) were tentatively identified from their characteristic UV spectra and chromatographic behavior, including retention characteristics, and were quantified using the quercetin calibration curve; their concentrations are therefore reported as quercetin equivalents. Kaempferol 3-O-glucoside and kaempferol 3-O-rutinoside were likewise tentatively identified and quantified using the kaempferol calibration curve, with concentrations reported as kaempferol equivalents. Concentrations were expressed as mg per 100 g dry weight of goji berries (mg/100 g DW). Each selected extract was analyzed in triplicate. Because the eight extracts constituted a targeted subset rather than an independent factorial experiment, the HPLC-DAD results were used primarily for descriptive, hypothesis-generating comparison of phenolic profiles.

### 3.9. Statistical Analysis

Results are presented as mean ± standard deviation (SD) of three independent experiments. The effects of drying pretreatment, extraction solvent, extraction temperature, and extraction time on LER, TPC, DPPH and ABTS were evaluated using multi-way factorial analysis of variance (ANOVA), including main effects and two-way interaction terms, in Python 3.14 using the statsmodels library (version 0.14.6). Statistical significance was accepted at *p* < 0.05. No post-hoc multiple-comparison procedure was applied; inferential interpretation was therefore based on the factorial ANOVA effects rather than pairwise comparisons between individual extraction variants. HPLC-DAD data from the targeted eight-extract subset were evaluated descriptively and were not subjected to inferential significance testing. Relationships among LER, TPC, DPPH, and ABTS responses were evaluated using Pearson correlation coefficients (r). Correlation analyses were performed for the complete dataset and separately according to drying pretreatment.

## 4. Conclusions

The present study demonstrates that liquid extract recovery (LER), total phenolic content (TPC), and antioxidant activity provide related but non-equivalent information about *L. barbarum* extraction. Across 160 extraction variants, the relationships between TPC and the DPPH and ABTS responses were only moderate or weak and varied with extraction conditions and berry pretreatment. LER likewise reflected phase-recovery behavior rather than conventional dry extract yield or phenolic extraction efficiency.

Targeted HPLC-DAD profiling of eight water and ethanol extracts further showed that contrasting technological extraction strategies were associated with different distributions of quantified phenolic compounds. Importantly, the 55 °C/48 h strategy produced substantially higher TPC, DPPH, and ABTS responses than the 70 °C/30 min infusion-like strategy, whereas the summed targeted HPLC phenolics differed by only 4.1%. This mismatch indicates that TPC and the targeted phenolic sum alone do not fully describe antioxidant behavior. Because the HPLC subset was evaluated descriptively and temperature and time changed simultaneously between the two strategies, these findings should not be interpreted as isolated factor effects or as proof of a causal contribution from individual compounds.

For food and nutraceutical applications, water and ethanol are the directly relevant solvent systems examined here, whereas tert-butanol and 1,4-dioxane served only as comparative media for probing extraction behavior. The results support combining technological process parameters, spectrophotometric assays, and chromatographic profiling when selecting extraction conditions and standardizing phenolic-rich goji ingredients. Future work should extend compositional coverage to non-phenolic reducing constituents and other antioxidant-active fractions, and should include product-quality attributes such as color when optimization is intended for practical food applications.

Overall, extraction strategy should be selected according to the intended product and target chemical profile rather than by maximizing TPC alone. The integrated analytical approach used here provides a framework for distinguishing process recovery, global reducing or radical-scavenging responses, and targeted phenolic composition in *L. barbarum* extracts.

## Figures and Tables

**Figure 1:**
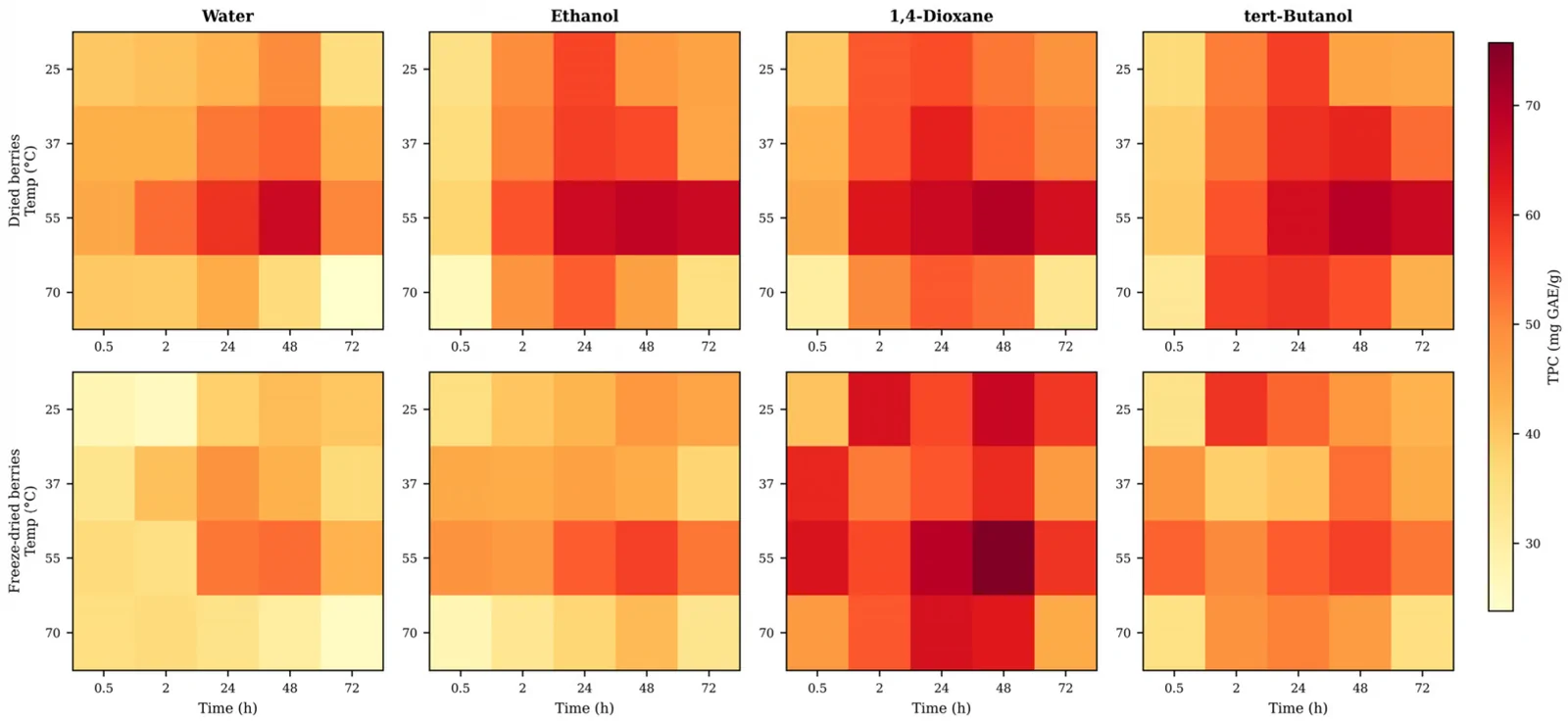
Total phenolic content (TPC, mg GAE/g DW) across 160 extraction variants, shown as a function of extraction temperature (°C) and time (h), faceted by drying pretreatment (rows) and solvent (columns). Color intensity reflects TPC magnitude.

**Figure 2:**
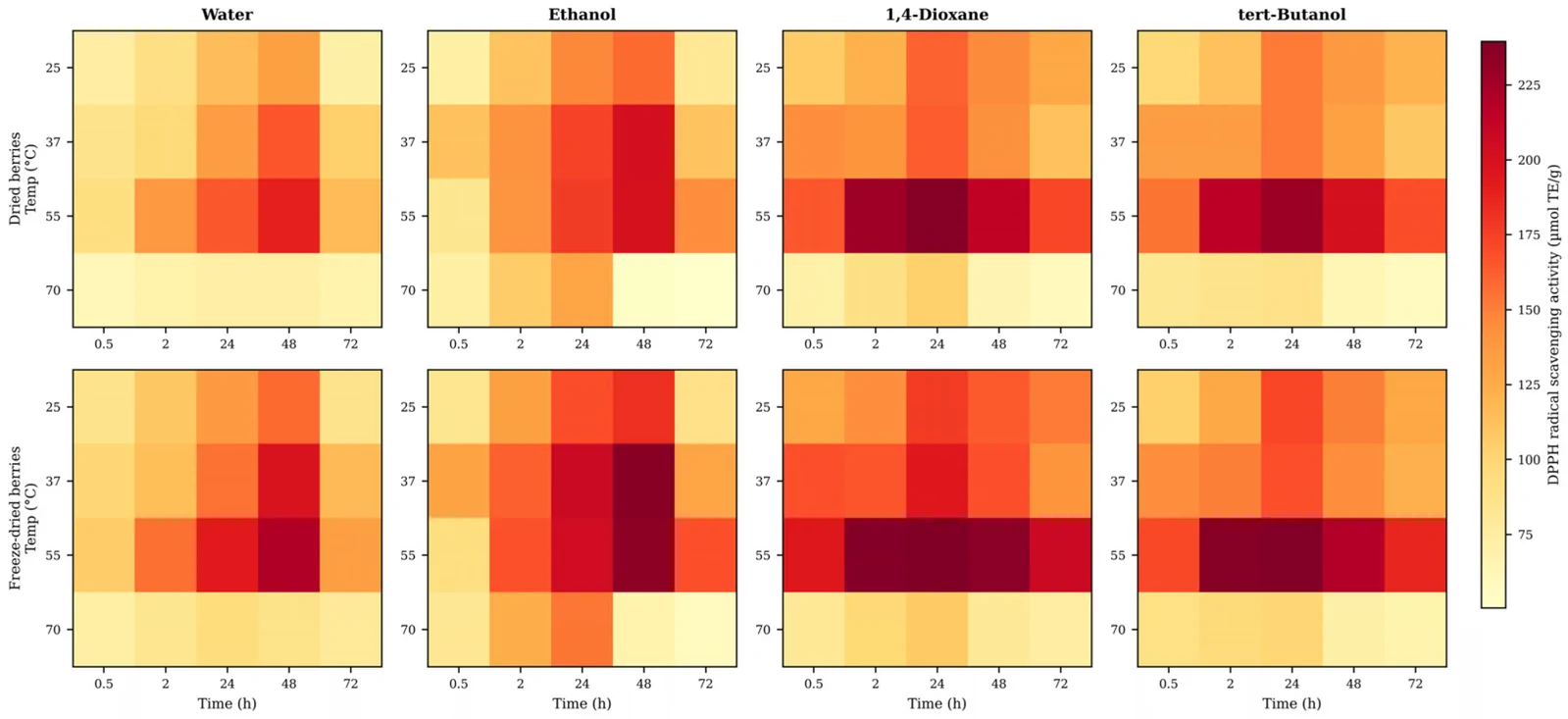
DPPH radical scavenging activity (µmol TE/g DW) across all 160 extraction variants, shown as a function of extraction temperature (°C) and time (h), faceted by drying pretreatment (rows) and solvent (columns). Color intensity reflects DPPH magnitude.

**Figure 3:**
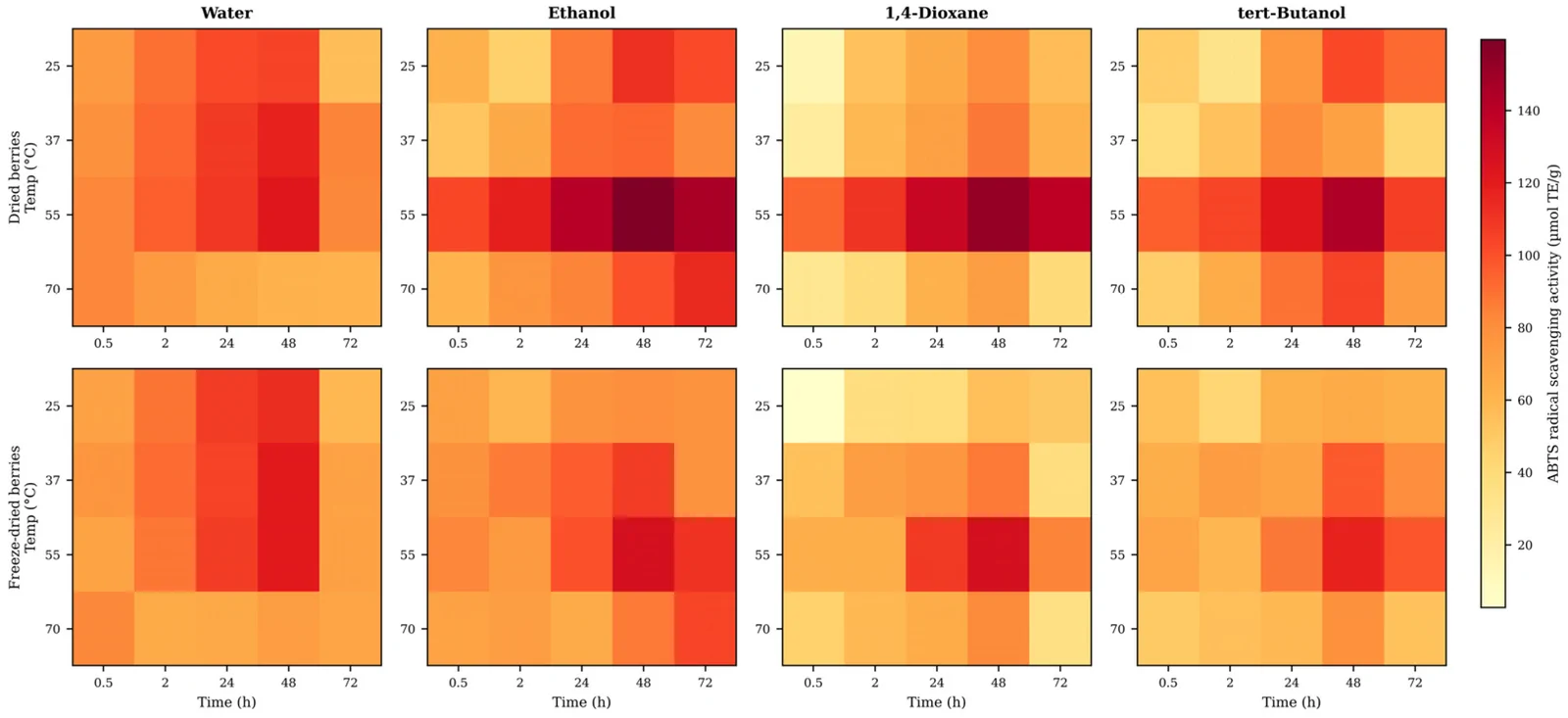
ABTS radical scavenging activity (µmol TE/g DW) across all 160 extraction variants, shown as a function of extraction temperature (°C) and time (h), faceted by drying pretreatment (rows) and solvent (columns). Color intensity reflects ABTS magnitude.

**Figure 4:**
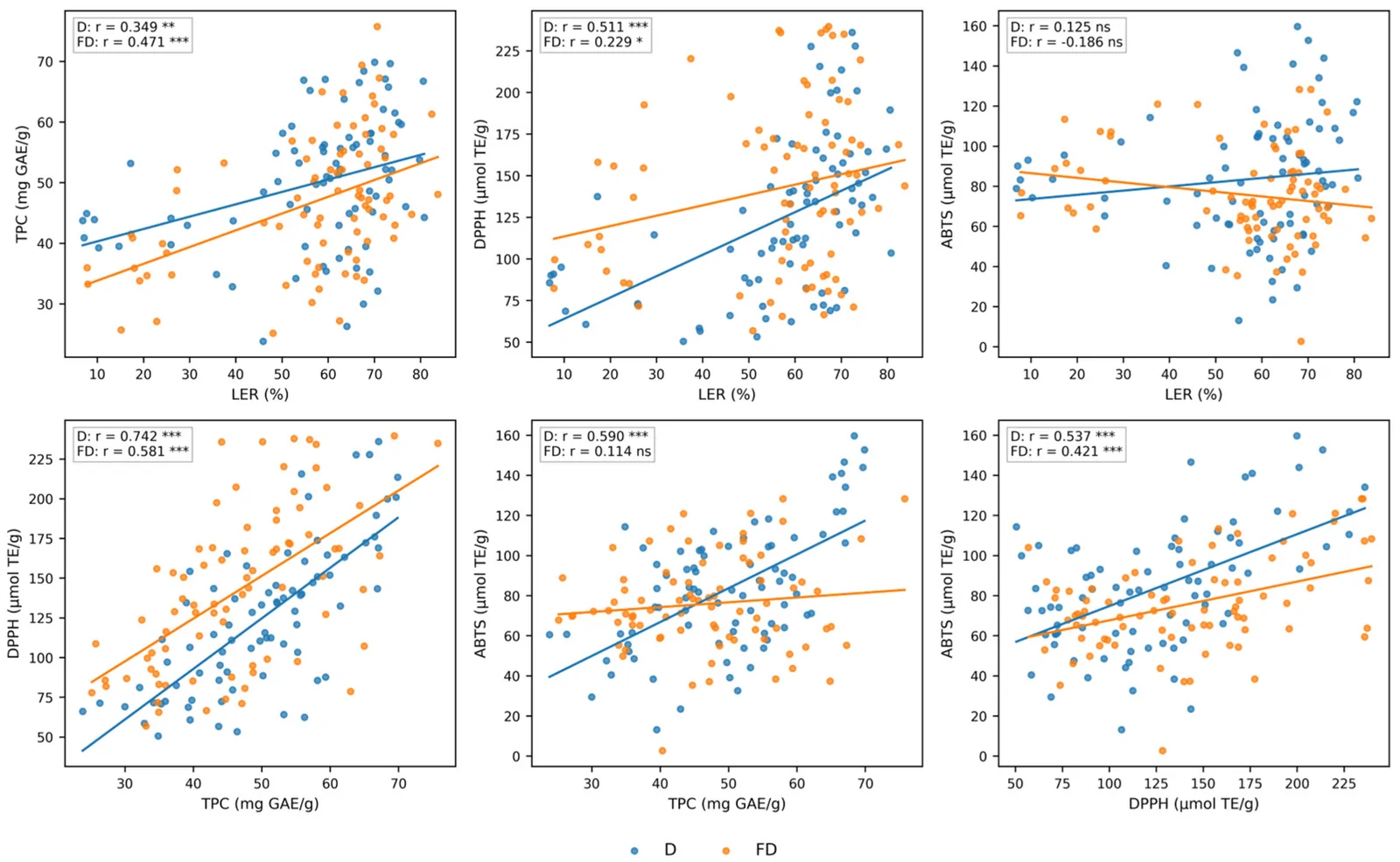
Pairwise scatter plots among LER, TPC, DPPH, and ABTS for all 160 extraction variants, stratified by drying pretreatment (D—dried, orange; FD—freeze-dried, teal), with separate linear trend lines and Pearson correlation coefficients for each group (*n* = 80 per group). * *p* < 0.05; ** *p* < 0.01; *** *p* < 0.001; ns = not significant.

**Figure 5:**
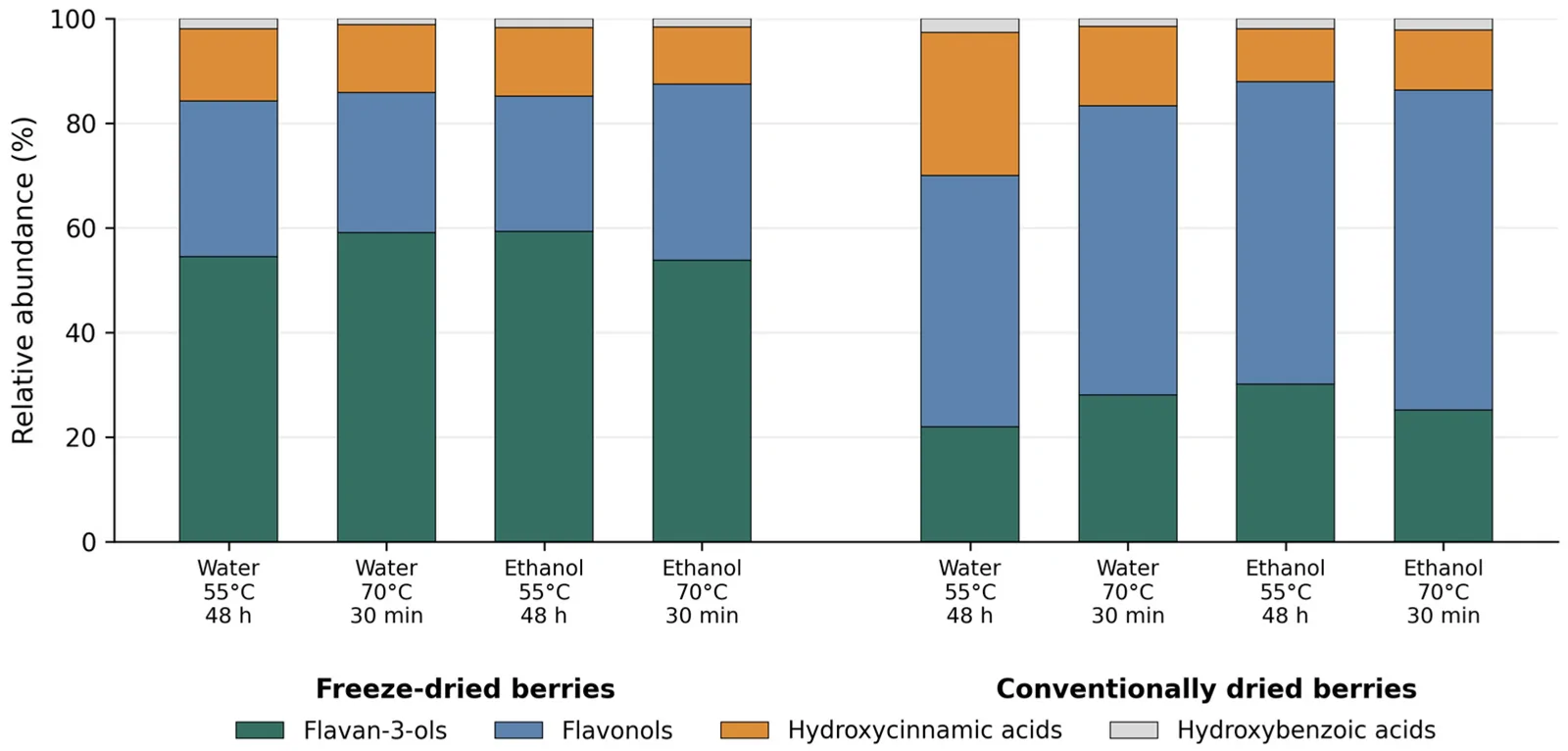
Relative abundance (%) of the four principal phenolic subclasses quantified by HPLC in the eight selected *L. barbarum* extracts. Trace apigenin, classified separately as a flavone, was excluded from the four-subclass percentage summary and is reported in Table 6.

**Table 1:** Factorial ANOVA evaluation of liquid extract recovery (Drying × Solvent × Temperature × Time, main effects and two-way interactions).

Effect	df	F	*p*
Drying	1	0.52	0.470 (n.s.)
Solvent	3	131.04	<0.001
Temperature	3	13.86	<0.001
Time	4	22.85	<0.001
Drying × Solvent	3	12.59	<0.001
Drying × Temperature	3	4.93	0.003
Drying × Time	4	6.36	<0.001
Solvent × Temperature	9	1.20	0.304 (n.s.)
Solvent × Time	12	14.15	<0.001
Temperature × Time	12	2.91	0.002

df—degrees of freedom; F—F-statistic (ratio of variance explained by the factor to residual variance; larger values indicate a stronger effect); *p*—significance level (*p* < 0.05 considered statistically significant); n.s.—not significant. Residual df 105.

**Table 2:** Mean liquid extract recovery by solvent type.

Solvent	Mean Filtrate Recovery (%)	Range (%)	SD	Key Observation
Water	35.93	6.84–80.79	24.60	lowest and by far the most variable recovery
70% Ethanol	60.71	35.80–78.09	8.08	high, considerably more stable than water
50% 1,4-dioxane	64.00	39.27–82.41	8.23	one of the highest recoveries obtained
50% tert-butanol	65.84	39.42–83.74	8.55	highest mean recovery

SD—standard deviation; *n* = 40 per solvent (160 extraction variants ÷ 4 solvents).

**Table 3:** Factorial ANOVA evaluation of total phenolic content (Drying × Solvent × Temperature × Time, main effects and two-way interactions).

Effect	df	F	*p*
Drying	1	18.89	<0.001
Solvent	3	60.40	<0.001
Temperature	3	61.98	<0.001
Time	4	48.60	<0.001
Drying × Solvent	3	16.44	<0.001
Drying × Temperature	3	1.77	0.157 (n.s.)
Drying × Time	4	6.91	<0.001
Solvent × Temperature	9	1.28	0.254 (n.s.)
Solvent × Time	12	1.31	0.224 (n.s.)
Temperature × Time	12	3.54	<0.001

df—degrees of freedom; F—F-statistic; *p*—significance level (*p* < 0.05 considered statistically significant); n.s.—not significant. Residual df 105.

**Table 4:** Pearson correlation matrix (r) among LER, TPC, DPPH, and ABTS for the complete dataset (*n* = 160).

	LER	TPC	DPPH	ABTS
LER	1.000	0.409 ***	0.353 ***	−0.005 ns
TPC	0.409 ***	1.000	0.611 ***	0.393 ***
DPPH	0.353 ***	0.611 ***	1.000	0.439 ***
ABTS	−0.005 ns	0.393 ***	0.439 ***	1.000

*** *p* < 0.001; ns = not significant. Diagonal values (1.000) represent each variable’s correlation with itself. LER—liquid extract recovery; TPC—total phenolic content; DPPH—2,2-diphenyl-1-picrylhydrazyl radical scavenging activity; ABTS—2,2′-azino-bis(3-ethylbenzothiazoline-6-sulfonic acid) radical scavenging activity.

**Table 5:** Pearson correlation coefficients (r) among LER, TPC, DPPH, and ABTS, stratified by drying pretreatment (*n* = 80 per group).

Correlation Pair	Dried	Freeze-Dried
TPC–DPPH	0.742 ***	0.581 ***
TPC–ABTS	0.590 ***	0.114 ns
DPPH–ABTS	0.537 ***	0.421 ***
LER–TPC	0.349 **	0.471 ***
LER–DPPH	0.511 ***	0.229 *
LER–ABTS	0.125 ns	–0.186 ns

* *p* < 0.05; ** *p* < 0.01; *** *p* < 0.001; ns = not significant. LER—liquid extract recovery; TPC—total phenolic content; DPPH—2,2-diphenyl-1-picrylhydrazyl radical scavenging activity; ABTS—2,2′-azino-bis(3-ethylbenzothiazoline-6-sulfonic acid) radical scavenging activity.

**Table 6:** HPLC-DAD phenolic fingerprint of the eight selected food-relevant *L. barbarum* extracts.

Phenolic Subclass/ Compound	Freeze-Dried Berries				Conventionally Dried Berries			
	EtOH 55 °C	EtOH 70 °C	Water 55 °C	Water 70 °C	EtOH 55 °C	EtOH 70 °C	Water 55 °C	Water 70 °C
Flavan-3-ols								
Catechin	8.30 ± 0.18	6.22 ± 0.12	9.40 ± 0.19	8.76 ± 0.16	8.58 ± 0.19	3.76 ± 0.11	3.84 ± 0.12	3.20 ± 0.10
Epicatechin	48.90 ± 0.98	38.84 ± 0.80	48.35 ± 0.96	53.35 ± 1.06	26.60 ± 0.53	22.27 ± 0.45	14.33 ± 0.29	23.15 ± 0.46
Hydroxybenzoic acids								
Gallic acid	nd	nd	nd	0.94 ± 0.02	nd	nd	nd	1.05 ± 0.02
Vanillic acid	nd	nd	0.37 ± 0.01	0.15 ± 0.01	nd	nd	0.41 ± 0.02	0.10 ± 0.01
Protocatechuic acid	nd	nd	1.68 ± 0.03	0.11 ± 0.01	nd	nd	1.71 ± 0.03	0.20 ± 0.01
4-Hydroxybenzoic acid	1.67 ± 0.03	1.33 ± 0.03	nd	nd	2.27 ± 0.05	2.20 ± 0.04	nd	nd
Hydroxycinnamic acids								
Caffeic acid	nd	nd	2.06 ± 0.04	1.89 ± 0.04	nd	nd	2.89 ± 0.06	2.10 ± 0.04
Ferulic acid	2.84 ± 0.06	2.64 ± 0.05	3.48 ± 0.07	2.06 ± 0.04	2.05 ± 0.04	1.83 ± 0.04	3.41 ± 0.07	1.57 ± 0.03
*p*-Coumaric acid	2.64 ± 0.05	0.86 ± 0.02	5.52 ± 0.11	2.75 ± 0.06	5.92 ± 0.12	6.64 ± 0.13	11.97 ± 0.24	5.98 ± 0.12
Chlorogenic acid	7.08 ± 0.14	5.67 ± 0.11	3.55 ± 0.10	6.97 ± 0.14	3.79 ± 0.10	3.41 ± 0.10	4.40 ± 0.10	4.63 ± 0.11
Flavonols								
Quercetin 3-O- Galactoside †	0.88 ± 0.02	0.95 ± 0.02	0.08 ± 0.02	0.72 ± 0.02	1.65 ± 0.03	1.45 ± 0.04	0.15 ± 0.01	0.69 ± 0.02
Quercetin 3-O-glucoside †	0.19 ± 0.01	0.10 ± 0.01	0.39 ± 0.01	0.17 ± 0.01	0.36 ± 0.02	0.39 ± 0.02	0.45 ± 0.01	0.52 ± 0.02
Quercetin dihexoside †	0.44 ± 0.01	0.41 ± 0.01	0.84 ± 0.02	0.42 ± 0.01	1.32 ± 0.02	1.30 ± 0.03	1.09 ± 0.02	1.12 ± 0.02
Rutin (quercetin 3-O-rutinoside) †	22.51 ± 0.50	25.07 ± 0.51	28.29 ± 0.60	25.30 ± 0.54	60.47 ± 1.21	56.94 ± 1.13	35.84 ± 0.72	46.71 ± 0.93
Kaempferol 3-O- Glucoside ‡	nd	nd	0.10 ± 0.01	nd	0.29 ± 0.01	0.22 ± 0.01	0.16 ± 0.01	0.14 ± 0.02
Kaempferol 3-O- Rutinoside ‡	1.00 ± 0.02	0.64 ± 0.02	0.93 ± 0.02	0.70 ± 0.01	1.55 ± 0.03	1.26 ± 0.03	0.99 ± 0.02	1.28 ± 0.03
Myricetin	nd	0.95 ± 0.02	0.93 ± 0.02	0.88 ± 0.03	1.81 ± 0.04	1.70 ± 0.03	1.20 ± 0.03	1.62 ± 0.03
Apigenin (flavone)	0.05 ± 0.01	0.03 ± 0.01	nd	nd	0.05 ± 0.01	0.04 ± 0.01	nd	nd

Values are expressed as mean ± SD (*n* = 3) and reported as mg/100 g dry weight (DW). nd, not detected. FD, freeze-dried berries; D, conventionally dried berries; EtOH, 70% (*v*/*v*) ethanol; Water, distilled water. † Tentatively identified quercetin glycosides, quantified using the quercetin calibration curve and reported as quercetin equivalents. ‡ Tentatively identified kaempferol glycosides, quantified using the kaempferol calibration curve and reported as kaempferol equivalents. Apigenin is classified as a flavone and is not included in the flavonol subclass totals.

**Table 7:** Comparison of the 55 °C/48 h and 70 °C/30 min technological extraction strategies for *L. barbarum* extracts.

Analytical Parameter	55 °C/48 h Strategy	Infusion-like Extraction (70 °C/30 min)	Relative Change * (%)
TPC (mg GAE/g DW)	61.57	31.95	+92.7
DPPH radical scavenging activity (µmol TE/g DW)	210.98	71.42	+195.4
ABTS radical scavenging activity (µmol TE/g DW)	132.75	74.29	+78.7
Σ targeted HPLC phenolics (mg/100 g DW)	100.50	96.59	+4.1

* Relative changes were calculated using the infusion-like extraction as the reference.

## Data Availability

The data presented in this study are available in the Supplementary Materials (Table S1).

## Supplementary Materials

The following supporting information can be downloaded at https://www.mdpi.com/article/10.3390/molecules31193441/s1, Table S1: Full dataset for 160 *Lycium barbarum* extraction variants, comprising liquid extract recovery (LER), total phenolic content (TPC), DPPH and ABTS radical scavenging activity for every combination of drying method, solvent, temperature and extraction time, together with descriptive summary tables by factor level and the correlation matrices reported in Table 4 and Table 5.
